# Supplementary material for: Association of adult caregiver depression with developmental disorder likelihood in Ugandan children perinatally exposed and unexposed to HIV
Source: Glob Ment Health (Camb). 2025 Oct 24;12:e120. doi: 10.1017/gmh.2025.10078 (PMC12641303; doi:10.1017/gmh.2025.10078)
Supplement: Awadu et al. supplementary material 1 — Awadu et al. supplementary material [file S2054425125100782sup001.docx]

**Table S1**: Developmental Disorder Probability Risk Scores among Dependent children over time, overall and within stratum of adult caregiver depression symptom level

|  |  |  |  |  | **One-way ANOVA (continuous variables)/Chi-square test (categorical variables)** |
| --- | --- | --- | --- | --- | --- |
| **Variables** | **Depression Level** |  |  |  |  |
|  |  | **Baseline** | **Month 6** | **Month 12** | **P-trend** |
|  |  | Mean (SD) | Mean (SD) | Mean (SD) |  |
| **Autism Spectrum Disorder** | Overall | -0.00 (0.99) | -0.16 (0.98) | -0.26 (0.98) | 0.0001 |
|  | Low (n=143) | -0.17 (1.00) | -0.30 (0.97) | -0.45 (0.88) |  |
|  | Moderate (n=306) | 0.01 (0.95) | -0.20 (0.90) | -0.24 (0.88) |  |
|  | High (n=155) | 0.13 (1.03) | 0.06 (1.12) | -0.17 (1.03) |  |
|  |  |  |  |  |  |
| **Attention Deficit Hyperactivity Disorder** | Overall | -0.05 (1.03) | -0.18 (1.00) | -0.28 (1.01) | <.0001 |
|  | Low (n=143) | -0.23 (1.03) | -0.46 (0.94) | -0.41 (0.97) |  |
|  | Moderate (n=306) | -0.02 (1.04) | -0.15 (0.94) | -0.25 (1.01) |  |
|  | High (n=155) | 0.06 (1.00) | 0.01 (1.11) | -0.27 (1.01) |  |
|  |  |  |  |  |  |
| **Emotional Behavior Disorder** | Overall | -0.03 (0.98) | -0.10 (1.02) | -0.16 (0.99) | <.0001 |
|  | Low (n=143) | -0.34 (0.93) | -0.35 (0.88) | -0.41 (0.86) |  |
|  | Moderate (n=306) | -0.03 (0.95) | -0.13 (0.98) | -0.19 (0.96) |  |
|  | High (n=155) | 0.26 (0.99) | 0.21 (1.16) | 0.03 (1.08) |  |
|  |  |  |  |  |  |
| **Resiliency Index** | Overall | 0.07 (1.04) | 0.22 (0.93) | 0.23 (0.92) | 0.1044 |
|  | Low (n=143) | 0.10 (1.14) | 0.30 (1.04) | 0.27 (0.96) |  |
|  | Moderate (n=306) | 0.02 (1.01) | 0.17 (0.91) | 0.18 (0.96) |  |
|  | High (n=155) | 0.13 (0.99) | 0.27 (0.87) | 0.29 (0.84) |  |
|  |  |  |  |  |  |
| **Functional Impairment** | Overall | -0.01 (1.02) | -0.16 (1.03) | -0.29 (0.98) | <.0001 |
|  | Low (n=143) | -0.27 (1.11) | -0.38 (1.08) | -0.47 (1.01) |  |
|  | Moderate (n=306) | -0.01 (0.97) | -0.19 (0.95) | -0.25 (0.94) |  |
|  | High (n=155) | 0.22 (0.98) | 0.11 (1.07) | -0.23 (1.02) |  |

All comparisons were derived from unadjusted means.
