# Supplementary material for: Association of adult caregiver depression with developmental disorder likelihood in Ugandan children perinatally exposed and unexposed to HIV
Source: Glob Ment Health (Camb). 2025 Oct 24;12:e120. doi: 10.1017/gmh.2025.10078 (PMC12641303; doi:10.1017/gmh.2025.10078)
Supplement: Awadu et al. supplementary material 2 — Awadu et al. supplementary material [file S2054425125100782sup002.docx]

**Table S2**: Time averaged association of Caregiver Depression level to attention deficit hyperactivity disorder (ADHD) and emotional behavioral disorder risk scores in their respective dependent children

| **Outcomes** | **Depression Level** | SMD (95% CI) | **Time * Depression Interaction** |
| --- | --- | --- | --- |
|  |  |  |  |
| **Attention Disorder Hyperactivity Disorder** |  |  |  |
|  | Low (n=143) | **-0.36 (-0.57, -0.15)** | 0.15 |
|  | Moderate (n=305) | -0.06 (-0.24,0.12) |  |
|  | High (n=155) | **Ref** |  |
|  |  |  |  |
| **Emotional Behavior Disorder** |  |  |  |
|  | Low (n=143) | **-0.57 (-0.78, -0.36)** | 0.26 |
|  | Moderate (n=305) | **-0.28 (-0.47, -0.09)** |  |
|  | High (n=155) | **Ref** |  |
